# After Coal: Affective-Temporal Processes of Belonging and Alienation in the Deindustrializing Nottinghamshire Coalfield, UK

**DOI:** 10.3389/fsoc.2020.00038

**Published:** 2020-05-28

**Authors:** Jay Emery

**Affiliations:** ^1^Department of Urban Studies and Planning, University of Sheffield, Sheffield, United Kingdom; ^2^School of Geography, Geology and the Environment, University of Leicester, Leicester, United Kingdom

**Keywords:** belonging, alienation, temporality, intergenerational transmission, affective memory, emotion, deindustrialization, class

## Abstract

This article advances conceptualizations of belonging and alienation among deindustrializing people toward (i) pluralistic temporal and (ii) affective processes. The focus is on belonging and alienation among a deindustrialized generation in the Nottinghamshire coalfield, UK, exploring how various affective-temporal processes mediate capacities, claims, and senses of belonging and alienation. Extant studies suggest that multiple temporal processes constitute deindustrialized places, particularly intergenerational transmissions, declarative memory, and place-histories. Recent work has explored the affective, emotional, and embodied dynamics of these temporal processes. While these literatures are insightful in locating affective and temporal processes of belonging, studies do not have much to say on the relational dynamics of affective-temporal processes in everyday becoming lives and experiences of deindustrializing places. The significance of foregrounding multiple affective-temporal processes of belonging and alienation is because of their relational nature. Advancing understandings of belonging is critical as a coherent sense of belonging is fundamental for individual and social well-being, and the loss of belonging, namely, alienation, informs how former industrial places are lived. Based on autoethnographic, interview and Observant Participation research with participants born between 1984 and 1994, I use ethnographic vignettes to delineate multiple relating affective-temporal processes of belonging and alienation of a generation that came after coal. The first vignette concerns the embodied and affective relationalities of intergenerational transmission and becoming in a deindustrialized world through the lens of masculinity, place and belonging. The second vignette examines nostalgic and traumatic shared declarative memories contingent of living through and with deindustrialization. The third vignette looks at intersections of place histories, silenced memory and local pride and shame, drawing out the significance of space and place to class-based experiences. Weaved through the stories are thematic threads of class, place, alienation, belonging, and temporality. Bringing these threads together, the paper then discusses the relationalities between issues covered, emphasizing the mutual contingencies between affective-temporal processes of belonging and alienation. I end by calling for shared affective-temporal processes of belonging and alienation to form the basis of a renewed solidarity, attenuation of alienation and a means to belong.

## Introduction

This article investigates temporal and affective processes of belonging and alienation under conditions of deindustrialization. The focus is on formations of belonging and alienation among a generation born between 1984 and 1994 into mining families and communities in the Nottinghamshire coalfield, UK. This ten-year period began with the fractious 1984–85 Miners' Strike, which preceded and precipitated the terminal decline of the British coal industry and led to the industrial ruination of the British coalfields. Memories and legacies of everyday life ordered around coalmining endure beyond colliery closures, evoking ambiguous, and conflicting affects in transforming deindustrializing presents. Although not fully understood, it is clear that temporal and affective dynamics are fundamental to senses of belonging (Game, 2001; Fenster, 2005; May, 2011; Nagel, 2011; Tomaney, 2015).

Literatures primarily focus on personal declarative memory—experiences that have been lived first-hand (Nagel, 2011; Lewicka, 2014; Lähdesmäki et al., 2016; May, 2016; Fivush et al., 2018). Declarative memory is but one, albeit multifaceted, temporal process that produces and mediates belonging. As an overarching temporal concept, memory itself involves multiple shared, intergenerational and personal processes and practices, ranging from the declarative and representational to the affective and embodied (Edensor, 2005; Degnen, 2016; Baraitser, 2017; May, 2017). These memory processes converge in the self to produce an individual's autobiographical narrative which has “functional significance” in producing and maintaining belonging, identity, and senses of the self (Brown and Reavey, 2015, p. 132). However, each of the myriad temporal processes are inherently contingent of spatial and social contexts that the individual finds themselves. In this important sense, certain temporal processes gain affective significance within certain spaces and times.

Studies of deindustrialization and social class have identified complex temporal processes mediating spaces of industrial ruination that are critical to the production and loss of belonging (Mah, 2012; Strangleman and Rhodes, 2014; High, 2018; Preece, 2020). Studies have acknowledged the affective dynamics of memory in the context of deindustrialization, drawing attention to the emotional intensities of loss, nostalgia, and bitterness attached to memories embedded in and evoked by landscapes and topographies of industrial ruination (Mah, 2012; Emery, 2019b). Relatedly, emphasis in recent years has been on what can be collectively termed “intergenerational transmissions,” past formations of everyday life that shape the present for those that lived those pasts as well as those that came after (Gordon, 1997; Mah, 2012; Meier, 2013; Walkerdine, 2015; Bright, 2016). Ambiguous in nature, emerging research concerns how intergenerational transmissions relate to affective dynamics of temporal processes, namely absence, embodiment, and the more-than-representational (Reay, 2005; Muehlebach and Shoshan, 2012; Degnen, 2013).

Similarly, there have been repeated calls to foreground affect in the study of belonging. Belonging is recurrently referred to as a state that “quivers” (Thrift, 2004, p. 57) with affective intensities, involves a range of emotional attachments and elicits myriad feelings (Mee and Wright, 2009; Wood and Waite, 2011; Yuval-Davis, 2011; Wright, 2015). The absence of belonging is also often expressed in negative affective terms as “feelings of loneliness, isolation, alienation, and displacement” (Antonsich, 2010, p. 649), and, following, I refer to loss and refusal of belonging as *alienation* here (Seamon, 2014). A coherent and stable sense of belonging is, thus, fundamental for individual and social well-being.

This article makes a conceptual contribution to belonging and alienation among deindustrializing people by advancing understandings in two interrelated directions. As signaled above, the first conceptual advancement is toward a pluralizing of the temporal processes bound up in the production and loss of belonging. Belonging is produced and mediated by myriad temporal processes, including intergenerational transmission, declarative memory and becoming, among many others (Rogaly and Taylor, 2009; May and Muir, 2015). The second conceptual advancement is an emphasis on the affective and embodied dynamics of these temporal processes. The importance of temporal processes to belonging is not so much their presence, accumulation or even contents, but the emotional attachments, bodily feelings, and affective meanings enlivened by encounters with them. The justification for proposing two advancements conjointly is the relationally constitutive nature of temporal *and* affective processes in the production, mediation, and attenuation of belonging. Apprehending affective-temporal relations is critical to understanding belonging and alienation among a deindustrialized generation as it is through these relational processes that we come to conceive who we are, the places we are from and where we belong and do not.

Approaching the affective, embodied, and emotional dynamics of temporalities as *affective-temporal processes*, this paper investigates the relationalities between intergenerational transmissions, declarative memories, place-histories, and becomings in the production and mediation of belonging and alienation. I suggest that these four relational affective-temporal processes, which are variably unlived, lived, and being lived through, deeply condition how belonging is claimed and denied for a generation that came after coal in the Nottinghamshire coalfield. Emerging from a focus on relationalities of affective-temporal processes is the fundamentality of place, class, and affective forms of experience, knowledge, and apprehension. In the Nottinghamshire coalfield, intergenerational transmissions manifest in a deindustrialized generation through embodied and affective intensities as pained and disdained bodies. Deindustrialization and neoliberal regeneration—individualizing discourses of social mobility, neoliberal regeneration programmes and austerity—has disrupted the capacity to fully adhere to our intergenerationally transmitted forms of belonging and identity so that we “become” in a transforming world that fragments our experience and trajectories of work and place (Brown and Reavey, 2015; Pain, 2019). Living with and through these socially fragmenting sociopolitical phases has led to competing claims over space and belonging, articulated through localized systems of knowing among bodies embedded in the histories of the coalfield. The capacity to anchor belonging in shared declarative memories as a strategy to cope with becoming in a fractured class landscape is obfuscated by the vulnerability of those memories, a consequence of growing up in conditions of deindustrialization. Moreover, use of the unlived past to produce autobiographical narratives is often occluded due to place-specific histories. A regime of silencing contentious pasts surrounding the Miners' Strike 1984–85, and the trade union splits and colliery closures that followed, prevails among the generation that directly experienced them (Paterson, 2014). These aspects of shared place-history are emotionally complex and difficult to communicate. They are, thus, regularly confined to personal memory without being declared or shared (Emery, 2019a). Such regimes of intergenerational transmission and silencing place-history can either enable belonging through the production of autobiographical narratives or inhibit belonging through gaps in autobiographical knowledge.

Critical for other deindustrialized areas, and research of them, the interrelations of these four affective-temporal processes sum up to a complex formation of belonging and alienation among a deindustrialized generation that is contingent of place, is multitemporal and evades easy recognition. Conceptualizing belonging and alienation towards a pluralized temporality and affect precludes simplistic explanations that solely highlight industrial job loss as the basis for loss of belonging and alienation. Deindustrialized spaces across the UK, Europe, and United States are continually linked with feelings of alienation and loss of belonging, not just in coalfield areas but in geographies previously dependent on, for example, fishing, steel, and many other forms of industrial production (Walkerdine and Jiménez, 2012; High, 2018; Silva, 2019; Preece, 2020). As the next section delineates, in former industrial areas the effects of deindustrialization unsettle and attenuate formations of, and capacities to, belong (Wacquant, 2016). Alongside alienation, the struggle to claim belonging when it is felt to be denied manifests in broader emotional intensities of bitterness and anger, operating at both personal and collective scales (Thorleifsson, 2016; Mishra, 2017; Harvey, 2018; Silva, 2019). Moreover, many deindustrialized geographies have received masses of targeted funding and initiatives, the British coalfields included, and, yet, belonging remains absent and alienation endemic. It is apparent, then, that the concept of belonging is both that regeneration policies toward social well-being have failed, and that belonging is deeply ambiguous. I propose that such future policies must first apprehend the complex relationships between temporality, affect and belonging.

Departing from Wright (2015, p. 392), I approach the affective-temporal processes of belonging through weak theory, which “sees things as open, entangled, connected, and in flux.” Remaining open to the potentialities of affective and temporal interrelations intends not to overdetermine conceptualizations in an emerging and interdisciplinary field of enquiry. Following, research involved complimentary methodologies associated with the study of affect and embodiment in Human Geography, where the impetus is “to get involved, to feel and care and be moved by what we are studying in the hope that our abstractions will be ‘less' abstract” (Spinney, 2015, p. 242; see also Hayes-Conroy and Hayes-Conroy, 2010; Dowling et al., 2017a,b). Principle among these are critical reflexive autoethnographies and using the researcher's personal histories and sensing and emoting body. Autoethnography is also common within studies of belonging, used to convey the deep emotional contradictions and intensities of belonging and alienation (Probyn, 1996; Butz and Besio, 2009; Hooks, 2009). As I am constituent of the generation under enquiry—born in 1986 into a mining family in the Nottinghamshire coalfield—I draw on my own experiences to document how affective-temporal processes intervene and mediate personal belongings and alienations. Toward a more representative analysis, I include data from 10 interviews and extensive Observant Participation collected as part of a larger project.

Following the explication of methods, I then use ethnographic vignettes to communicate (some) of the complex ways affective-temporal processes of belonging are lived for this deindustrialized generation, covering broad experiential contexts, and lived histories. The intention is not narrowly-focused, fine-grained analyses, but to disentangle affective-temporal processes of belonging and alienation at the same time as retaining, and emphasizing, their relational and everyday contingencies (Stewart, 2007; Cameron, 2012; Lorimer and Parr, 2014). The first vignette concerns the embodied and affective relationalities of intergenerational transmission and becoming in a deindustrialized world through the lens of masculinity, place, and belonging. The second vignette examines nostalgic and traumatic shared declarative memories contingent of living through and with deindustrialization. The third vignette looks at intersections of place histories, silenced memory and local pride and shame, drawing out the significance of space and place to class-based experiences. In the section that follows the vignettes I will discuss the relationalities between issues covered, emphasizing the mutual contingencies between affective-temporal processes of belonging and alienation. I first want to articulate further what I mean by affective-temporal processes and their importance to understandings of belonging among deindustrialized generations.

## Affective-Temporal Processes, Deindustrialization and Deindustrialized Generations

Studies of deindustrialization have recently centered on “intergenerational transmissions,” “social hauntings,” “legacies,” and “half-lives” (Mah, 2012; Walkerdine, 2015; Bright, 2016; Linkon, 2018; Warren, 2018). Although more particularized in their individual compositions, these concepts all index how industrial pasts condition how the deindustrialized present is experienced and encountered. Walkerdine (2015) suggests:

We can consider the intergenerational transmission of class as an interlinked set of hauntings in which discourses, practices, policies, and events are experienced and felt in the bodies of both those who experienced them *as well as those who followed them* (p. 172, emphasis added).

Research has identified various means that the intergenerational past—unlived for the generation in question for this article—emerges and is transmitted (Mah, 2012; Walkerdine and Jiménez, 2012; Hill, 2013). For instance, the industrial past emerges through testimony, declarative and collaborative remembering, landscapes, cultural representations, and practices and material cultures (Emery, 2019b). Emergences of the past are always relational and constitutive of each other, whereby, for example, landscapes evoke memories in older generations that then form the basis for collaborative remembering with their children (Walley, 2013; Pasupathi and Wainryb, 2018). As well as representational forms, there are affective and embodied dimensions to intergenerational transmissions in deindustrializing spaces, operating relationally with representational forms (Edensor, 2005). Material cultures from past industrial communities stimulate affective atmospheres, sensory memories of taste, smell, sound, touch and sight and evoke embodied, and emotional memories of past ways of being (Lucas and Buzzanell, 2011; Hill, 2013; Meier, 2013). Further, vivid and imagined sensory memories of erased industrial workplaces, despite material absence, are still very much present in the geographical imaginations and spatial memories of long-term residents. Imaginaries of erased or repurposed landscapes and topographies are superimposed on the new economies that replaced them. Erased material forms also remain present through the embodied practices and performances they helped engender, as former industrial communities navigate deindustrialization (Blokland, 2001).

Generations growing up in deindustrializing spaces seek to resist and make sense of industrial ruination through reference to intergenerational transmissions, with values, performances, behaviors, and social relations being historically constituted by industrial formations (Rhodes, 2013). The unlived industrial past mediates experiential contexts of employment (McDowell, 2003; Nixon, 2009; Walkerdine and Jiménez, 2012; Bennett, 2015b), education and school (Bright, 2012; Bathmaker et al., 2013; Ward, 2014), place (Nayak, 2003, 2006; Bright, 2016), and identity (Rhodes, 2013). A focus of the literature has been on how valued male occupational identities remain rooted in industrial forms of labor (High, 2003; MacKenzie et al., 2006; Walkerdine, 2010; Ward, 2014; Clarke, 2015, 2017). With labor market shifts from industrial work to service-based employment “industrial citizenship” has been fractured and men from industrial families struggle to form coherent senses of identity and belonging around what are considered locally to be feminine jobs (Strangleman, 2015). Lack of opportunity to perform masculinity through heavy labor employment has led to expressions of manhood to (re)center on excessive drinking, football and distinct gender significations (Nayak, 2003, 2006; Ward, 2014; McIvor, 2017). Little work has been conducted on women from industrial families, however, young women's socioeconomic experiences are also shaped by industrial legacies by circumscribing “what can be thought, said and done” (Bennett, 2015b, p. 1290; see also Taylor, 2012).

While conditioned by intergenerational transmissions, we must not overstate the hysteresis of deindustrializing places. Deindustrialized generations, and the spaces they occupy, are in continual everyday processes of becoming, evident in the material decay and regeneration that has taken place since the closure of industrial workplaces and production (Stewart, 1996; Whatmore, 2006; Pink, 2012; Hill, 2013; Degnen, 2016). For a deindustrialized generation, encounters with pasts that predate them happen at the same time as we become through sociopolitical policies, events, and transformations. For the deindustrialized generation in the Nottinghamshire coalfield, their “historicity of lived experiences” (Tilley, 1994, p. 23) has been shaped by several sociopolitical policy phases. First among these is the neoliberal discourse of individualized aspiration that has dominated Western societies since the 1980s (Bright, 2011; Ingram, 2011; Bright, 2012a, Bright, 2016b). Additionally, from 1998 to 2010—when the deindustrialized generation in this article were transitioning to adulthood—Labour Party governments initiated a programme of neoliberal regeneration in the coalfields areas involving replacing lost relatively high paid, stable industrial employment with low paid, precarious, and menial jobs in service industries (Shildrick et al., 2012; Foden et al., 2014). Finally, austerity measures over the last decade have disproportionately impacted former industrial areas, eroding further social support systems and entrenching social inequalities and its consequent material and environmental decline (Beatty and Fothergill, 2016).

These sociopolitical policies amount to a sustained process of dispossession inflicted on deindustrialized places and people, that has led to fragmented relationships between people and place. The narrative instilled into deindustrialized generations over successive sociopolitical phases is that we should reject the deindustrialized places and class we are from and should strive for individual social and spatial mobility (Tyler, 2013; Wacquant, 2016; Crossley, 2017; Nayak, 2019). Notwithstanding the punitive and stigmatizing measures facing deindustrialized people who “fail” or reject this agenda, upwardly mobile individuals with working-class backgrounds can experience intense feelings of “unease, anxiety, and dislocation” (Friedman, 2016, p. 130) engendered by social mobility and dwelling within middle-class spaces such as university or workplaces (Ingram, 2011; Taylor, 2012; Bathmaker et al., 2013).

Moreover, the affective-temporal process of becoming through these phases has been encoded in declarative memories of a deindustrialized generation (Pain, 2019). While declarative memory is critical to belonging, it is contingent on the histories available to remember and construct autobiographical narratives. Through processes of becoming under a regime of neoliberal regeneration, decline and false social mobility deindustrialized generations accumulate experiences that translate into declarative memories, emotional repertoires, and bodily dispositions that can alienate, as well as anchor belonging (Anderson, 2014).

Not all deindustrialized people have been maladaptive to recent transformations and many navigate tensions between pasts and presents successfully (Bathmaker et al., 2013; Loveday, 2014; Roberts, 2014). Place and place-histories have proved restorative where deindustrialized generations can draw upon proud histories to sustain themselves in uncertain or fractious times and place remains a primary attachment for belonging (Walley, 2013; Bright, 2016; Emery, 2018; Preece, 2020). Stephenson and Wray (2005) have documented “emotional regeneration” in the north eastern coalfield of England, achieved through engaging with cultural inheritances, specifically by the commissioning, display, and maintenance of union banners, which act as the “symbolic and representational heart of [their] village” (Ibid, p. 180). More broadly, it is often suggested that historical awareness and heritage has a therapeutic capacity to enhance belonging and process change in deindustrialized communities (Linkon and Russo, 2002). Walley (2013, p. 22) describes this process, from a personal perspective, as like “scratching an itch, or salving a wound.” This also has a clinical basis, with social and cognitive psychologists arguing that practices of collaborative remembering—the co-production of shared narratives on the past between active participants—facilitates senses of belonging and shared identity (Fivush et al., 2018). However, as will be revisited, how place and place-histories are (re)produced, communicated, and curated is important in places where the past is deeply contested, such as the Nottinghamshire coalfield.

Collectively, the above literatures have been useful in evidencing the relational dynamics between industrial legacies and memories of deindustrialization. In summary, the lives of a deindustrialized generation are experienced through multiple and relational affective-temporal processes converging in and evoked by places, bodies, and materialities. Apparent is the extent in which affective-temporal processes are embedded in practices, performances and embodiments of everyday life, imbuing experiences of work, education, place, and social relations (Blokland, 2001; Connerton, 2011; Baraitser, 2017). However, studies of belonging do not have much to say explicitly on how affective-temporal processes relate and entangle to mediate senses of belonging and alienation (Degnen, 2013; May, 2016; Baraitser, 2017). As will be documented, everyday exposures to relational affective-temporal processes of intergenerational transmission, becoming, declarative memory and place-history constitutes and intervenes in the capacity to belong and be alienated (Bennett, 2014b; Emery, 2019a). First it is necessary to detail theoretical and methodological approaches taken.

## Conceiving and Apprehending Affective-Temporal Processes of Belonging

Couched in the affective turn, belonging is a constellation of emotional attunements, affective attachments, and embodied feelings, and there is increasing awareness of the affective and emotional relationalities of belonging and temporality (Bennett, 2009, 2014b; Mee and Wright, 2009; Jones, 2011; Wood and Waite, 2011; Brown and Reavey, 2014). Not seeking to reify concepts of belonging, or its affective and temporal contingencies, following Wright (2015), I propose a weak theory approach to belonging (see also: Sedgwick, 1997, 2003; Anderson, 2014). Weak theory resists strong theory's “tendency to beat its objects into submission to its dreamy arguments” (Stewart, 2013, p. 284), allowing room “to wonder where [objects of study] might go and what potential modes of knowing, relating, and attending to things are already somehow present in them as a potential or resonance” (Stewart, 2008, p. 73). Further, “weak theory also points to the affective domain, to the myriad more-than-human processes of attunement and attachment through which belongings are constituted” (Wright, 2015, p. 392). Approaching belonging through weak theory means attending to the sometimes amorphous aspects of affective life, particularly the affective atmospheres or “structures of feeling” (Williams, 1977) that envelope and constitute spaces, localized systems of knowing and relating and the embodied, non-declarative, spaces inherent in human and more-than-human interactions (Anderson, 2014).

I do not, however, conceive affect as some purely non-representational or unreachable other (Anderson, 2014; Fox, 2015). Rather than an overdetermination of its uncanny dimensions, affect frameworks allows us to consider several interrelated and relevant conceptualizations of embodiment, emotion, and the atmospheric. In my reading, affect is part of wider flows of signification within recognizable socio-spatial processes (Burkitt, 2019). Affect and emotion, much like memory, are personally intimate, but are also socially produced, felt in common and rendered intelligible by social systems of knowing. A room can be characterized by tension, a nation can be in mourning, ages of anxiety, paranoia and emergency periodize history, and institutions can manifest cultures of hate or fear. Further, evidenced below, shared affective conditions also take place at localized geographical levels and spaces can be occupied by competing senses of atmosphere that evoke disparate emotional states. As Burkitt (2019, p. 55) argues, emotions “are part of human activity and are experiences that emerge out of specific situations in which we are related to other people and things in a socially meaningful way.” Shared affective conditions that exist outside human bodies, such as atmospheres, collective moods or structures of feeling, manifest in the emotional, and embodied registers of the human (Anderson, 2014). And human bodies, in turn, produce those shared affective conditions, co-acting relationally with their human, material, symbolic, and representational geographies. Social scientists concerned with emotion and affect engage with the social, material, discursive, and atmospheric through closer attunement to the sometimes hidden, yet fundamental, aspects of experience (Wetherell, 2012, 2013; Wetherell et al., 2015). For me, “becoming sentient to a world's work, bodies, rhythms, and ways of being in noise and light and space” (Stewart, 2011, p. 445) merely involves recalibrating existing methodological approaches toward more embodied, emotional, and critically reflexive research sensibilities (Hoggett et al., 2010).

The use of the critically reflexive self and the emoting, sensing body is central to affective methodologies, although is particularly open to claims of self-interest, indulgence, and atypicality (Jones, 2011, 2015; Horton and Kraftl, 2012; Mah, 2014; Shaw et al., 2015). What this work achieves, however, are substantive accounts of how temporal processes—particularly remembering declarative memories—are deeply affective and emotional and a practice tied to landscapes, materialities, and other evocations, and situated within contemporary and changeable moments and contexts (Ellis and Bochner, 2000; Butz and Besio, 2009; Moriarty, 2013; Bondi, 2014a; Jones, 2015; Wylie, 2017). Relatedly, declarative memories emerge from *shared* spatial, temporal, and affective processes (May, 2013). Being from a mining family in the Nottinghamshire coalfield, I am from the same intergenerational shared spaces as those under enquiry. Drawing on the concept of habitus, Ingram (2011, p. 289) states “when people share similar life experiences by, for example, growing up in a particular working-class neighborhood, they acquire dispositions in line with those of their families and neighbors. Moreover, scholars have recently sought an “expanded view” (Brown and Reavey, 2015) of memory, thinking more relationally between personal, familial, and social memory and highlighting the collective nature of emplaced recollections of shared pasts (Hoskins, 2016, 2018). Returned to in the final discussion, there are limitations to autoethnographic enquiry, both applicable to all social research and specific to this study. In a general sense the ways that affects and emotions are experienced, condition, and read is affected by various other subjectivities, identifications and lived histories of the body. My emotional and lived experiences have been conditioned by my white male positionality, and this positionality will also impact my capacity to interpret others” emotional and lived experiences. More specific to this research, there are also concerns that my enhanced knowledge of local histories, and my position to that history, renders me unrepresentative of others from the same generation. However, as will later be argued, individualized trajectories in a deindustrialized geography make everyone unrepresentative to some degree. As the vignettes detail, my declarative memories and becomings are regularly aligned and entangled with those of my generation who all share experiences structured by deindustrialization and the histories of the Nottinghamshire coalfield. Autoethnography is used here to elucidate emotionally difficult experiences from these shared pasts and presents that are often withheld in interviews.

There are limits to what is retrievable through interviews, especially given the emotionally intense subject focus here which can be difficult to express authentically (de Boise and Hearn, 2017). I do, however, draw from interviews conducted between May 2016 and March 2018 with five women and five men born between 1984 and 1994. The participants all have genealogies embedded in the Nottinghamshire coalfield and the coal industry, yet, all have different experiences of place, becoming, work and social relations. Questioning involved aspects of life-history and psychosocial interviewing, both premising that our present-day affective selves are products of our histories and memories (Wengraf, 2000; Jackson and Russell, 2010; Walkerdine, 2010, 2016; Hollway and Jefferson, 2013). Analysis paid close attention to the whole of the interview as a relational and situated process, foregrounding the atmospheres, contexts, and emotional dialogues of the interview setting beyond solely speech (Hollway and Jefferson, 2013; Bondi, 2014b; Dowling et al., 2016). Collaborative remembering was a notable feature of the interviews. Although I did not know the participants prior to research, during interviews we would arrive at shared experiences of the communities we grew up in and developed dialogues around familiar subjects, such as school, stories our parents, and grandparents told us and nights out in our localities and in Mansfield, the main urban center in the Nottinghamshire coalfield. In some instances, sharing reminiscences of growing up and experiencing the Nottinghamshire coalfield put participants at ease, but also made some wary of elaborating on emotive subjects. I propose that this reticence came from a supposed likelihood that we may have mutual friends or acquaintances and was evident in the persistent questioning into my own past, where I grew up and what schools I attended.

In addition to interviews, I engaged in 2 years of Observant Participation alone or with friends in Mansfield and its surrounding former coalmining villages. An explorative and reactive Observant Participation replicates how life is lived and seeks to capture some of the temporal and affective encounters that take place in everyday life (Law, 2004; Stewart, 2005; Pink, 2012; Plows, 2018). Similar to Participant Observation, Observant Participation aims to “move beyond surface appearances and study what they, rather than their informants, think is important” (Moeran, 2007, p. 14) and is well-suited for researchers with pre-existing knowledge of the research field. Observant Participation was conducted day and night and took place in streets, public houses, cafes, Miners' Welfares, football matches, takeaways, restaurants, shops, and nightclubs and involved a focus on everyday processes, interactions and performances of people around me, noting what was being said and what was not (Bennett et al., 2015). It was sometimes a confusing experience shifting positionalities from being of somewhere, and embedded in its spaces, to conducting research of them, leading to conflicts of belonging and alienation that were representative of the effects of deindustrialization itself, and explored in the first vignette.

## Intergenerational Transmissions, Pained Bodies and Pained Places

My dad used to have a swelling of fluid below his proper knee, prepatellar bursitis accumulated from sustained kneeling. As kids, my siblings and I called it his third knee and took grotesque pleasure in poking it. I can still sense the texture and sponginess of it now. My grandad's body, too, seemed totally inscribed by physical labor. He wore a cervical collar most evenings to ameliorate the piercing pain of arthritis acquired by years of climbing through dark underground tunnels. My siblings and I liked to wear my grandad's collar, finding it hilarious to walk around pretending to be Frankenstein's monster. He also had the end of his finger chopped off at work so that his nail grew over the top. Although my grandad died some years ago, I can still picture the curve of his nail and the sound it made tapping on his cup of tea.

Laboring bodies hurt. Often because of accidents, but just as much as an unavoidable consequence of the contortions, abrasions, and erosions that they must do to perform their job. Women in the Nottinghamshire coalfield are not excluded from this injurious process, but it is the stories and bodies of men—of miners and mining—that dominate. The injured bodies of our male family members are fundamental to how we perceive them: physical, laboring, marked by arduous work. Participants all had dads or grandads who had impairments from their labor, and they have affective and embodied childhood memories of being fascinated by coal tattoos, or intrigued by missing fingers or limbs, or empathetic to wheezing lungs. Injured men and women surrounded us beyond the home and family as well. It seemed ordinary to see middle-aged men using walking sticks, for instance. We were habituated in home and community to occupational afflictions and stories about accidents suffered within the harsh conditions of the colliery. Miners and ex-miners would sit in homes, pubs, and Miners' Welfares tapping parts of their body that had befallen some excruciating incident or pulling aside trouser legs, shirt sleeves, or collars to reveal scars. As they spoke, we would be surrounded by material reminders of the mining days. Commemorative plates, coal carvings, ornamental miner's lamps, and pin badges acted as memory prompts and symbolic reminders for more remembering. We came to know where we were and from through these materialized and embodied intergenerational stories, gifted to us as transmissions of how work should impart itself on us and others, and how our bodies should feel to belong (Bennett, 2014a, 2015a,b).

For 5 years before going to university, and in the summers as an undergraduate, I worked in the construction industry. On one occasion my right leg slipped through some scaffolding while I was carrying some building materials. The severe pain was temporary, however the numbness continued for several weeks after and, when the gashes healed, a slight indentation was left on my thigh. Sometimes, when I am thinking about the different type of work I do now, how different it is to the physical labor I did before and that most of my male family members and childhood friends do, I feel a sensation where my injury had been. I have a conflicting desire for the indentation to be bigger, for the pain to still be present, so that I can share in the telling of stories and memories that men I know engage in, sat trading stories of injury, pain and labor. It is a sensation I felt while talking to Stephen and Simon about their jobs.

Stephen is a plasterer and, since the 2008 financial crisis, has been employed by “six or seven different firms,” as the companies either went bankrupt or had to lay-off workers. Nonetheless, Stephen sees his job as a “proper job.” It is physical, regularly hurts him and after work he feels physically drained. It is how his dad and grandfather would have “felt after a shift at the pit,” and, in managing to align with intergenerationally transmitted valuations of meaningful work, these embodied lineages connect him to male ancestors. Stephen's body feels as if it belongs within space and time. There is a cognitive and affective barrier between Stephen and me as we speak. I know that Stephen does not think my job is a “proper job,” and I know that if I mentioned that I once had a “proper job” it would only intensify Stephen's disdain for me. I received a similar reaction from Simon, a warehouse operative at a large company built on reclaimed land where the colliery once was.

Simon hates his job. It is boring and he “don't get paid much.” If the colliery was still open he would be “down there in a shot.” His dad, grandad and, maybe, his great-grandad (Simon's not sure) all worked there but Simon was disinherited from his patrilineal tradition when the colliery closed in 1989 (Preece, 2020). He must work though and would never go on benefits or join the “Bad Back Brigade”—former miners who are suspected of feigning bad backs in order to avoid work and live on incapacity payments. Fear of joining this dishonorable “brigade” ensures that Simon works through illness and injury in a job that alienates him daily. It is clear that Simon is resentful, probably of me. He mentioned three times during our hour-long interview that I was exempted from the alienations he was struggling to articulate because I, in Simon's words, “went to uni.”

Higher education is indeed a tangible route for a deindustrialized generation and three participants, additional to me, had attended university. However, intensities of alienation were continually forced to the surface for those that attended, in moments that stick with them (Massey, 2011). Lauren still remembers vividly and affectively the “chav nights” at her university when fellow students came to her room to ask if she had any “chavvy” clothes they could borrow: “I just thought, ‘What, cause I'm from Mansfield and I've got this accent?' I mean, who d' they think they are? I jus' felt so awkward for ages after that. I jus' thought, ‘aww God, where am I ‘ere?” I, too, had similar experiences. In my first year at an “elite” university I attended a varsity rugby game. The atmosphere was quite good-natured as we squashed together on the benches, until it transformed, as the chants from “our” side began to rouse. Those I was sat with, that I had lived with for the previous 8 months, joined in as a chorus started: “Your dad works for my dad! Your dad works for my dad!” The next: “We pay your benefits! We pay your benefits!” Surrounded by the voluminous noise, the feeling of alienation swelled. The felt intensity derived from the unexpectedness, and the speed in which an affective atmosphere can change in an instant, from one imbued and facilitative of communal belongings to one that excludes. The chants continued throughout and each time the feeling of alienation, lingering in the background as a knot around the stomach, loosened and tightened along with chants. The memory is still drenched with conflicting emotions—resentment, defiance, shame.

Simon and Stephen are justified in their assessments though. I am symbolically, if not economically, distanced to them as a result of our different deindustrialized trajectories. After our interviews, I wondered if either Simon or Stephen are “typical Mansfield lads.”^1^ I knew exactly the type of man who Lauren, Kate and Claire meant when they each, in separate conversations, mentioned “your typical Mansfield lad.” But when we tried to articulate him, to flesh out the typical Mansfield lad's character, the way he looks, his disposition, his behavior, and opinions, we could not quite pin it down. He evaded us although we knew him so well and we laughed together about how a character so known to us could simultaneously be so elusive. The same phenomenon pervades conversations in the Nottinghamshire coalfield all the time. People flippantly state that someone is just “your typical Mansfield lad.” We are all convinced of the meanings contained in these shorthand phrases. We are certain that there is a shared understanding, although this must be taken-for-granted as we cannot put it into words. We just feel as if we *know* because we all grew up there or in nearby mining villages.

Claire is married to someone who she describes as “just your typical Mansfield lad.” Claire says this with a sense of uncertainty, absent of pride, and understating her own love for her partner. It is as if she expects me to think less of her and her husband and is insulating herself against stigmatizing evaluations. Claire quickly backtracks and qualifies previous statements by suggesting that “other people could think he is [a ‘typical Mansfield lad'], but he in't really.” It seems that no one is, at the same time as them existing in droves. The “typical Mansfield lad” is a term used to deride and alienate, to signify negative masculine attributes and performances, that induce negatively valenced affects, to make you feel disdain and discomfort. He is perhaps someone “still lives with their mum but has this ‘amazing' car on finance” (Lauren). Or he is misogynistic, “grabbing your arse on a night out” (Kate), wears designer clothing associated with football hooligans, has his priorities wrong and fights a lot. These embodied performances generate Mansfield's volatile affective atmospheres, atmospheres that alienate and make people feel anxious and wary of violence and abuse.

Participants squirmed when probed on their feelings toward “typical Mansfield lads.” They felt a bit “judgy” (Lauren), as if they were displaying some prejudice and breaking an invisible code of solidarity with a place they know has suffered and that they have suffered with. Mansfield used to have a thriving nightlife, but people have stopped going out in the town. Partly, participants claimed, because the presence of “typical Mansfield lads” puts them off. Prejudice toward people from similar class backgrounds and places rankles with me, but I am just as bad (Wacquant, 2016). I did not particularly want to go on nights out in Mansfield for this research because I knew what to expect, and sometimes think Mansfield is, like Dan says, “a shithole.” Yet, certain men adopt embodied behaviors and dispositions associated with “typical Mansfield lads” partly because the resources to belong are severely lacking in other contexts—particularly, as with Stephen and Simon, employment and work. The more nuanced mimetic performances and behaviors that Lauren, Kate and Claire dislike so much are attempts to claim spaces to belong. Through doing so the “typical Mansfield lad” and those from a similar class and family background exclude each other based on feelings evoked in and by each other and the atmospheres they generate in the spaces they occupy. Places such as Mansfield then become contested spaces with competing claims over who belong in and to them by opposing sides both comprised of a same deindustrialized generation. These conflicts have troubled relationships throughout our lives and compromise declarative memories, with impacts on formations of belonging.

## Lived Memory, Living Trauma and Misrecognised Nostalgia

Matt thinks that he was in a minority in his school year, so he was surprised he was not bullied as a result. He was into different things—skating culture and the clothes and music associated with it. There were enough of these types around to form their own small group. They were marginalized from the more dominant majority, and regularly felt threatened by them, but this marginalization, for Matt at least, stopped short of “out-right bullying.” It helped that his father was quite well-known in the colliery village where they lived and had taught Matt how to handle himself in a fight. Matt, hungover, being interviewed by a relative stranger, strikes for balance and reflects that “everyone got bullied a little bit in schools like ours though, didn't they?”

“What schools are they?” I asked. Even though we went to different schools, I already had a sense of what Matt's answer was to be.

“Schools ‘round Mansfield. They were all a bit … tough, I guess,” replies Matt.

Secondary school was certainly tough for Laura when she was bullied in the late 1990s. She still remembers vividly the beating she endured on the playing fields on her way home one day. Her bullies thought she was “stuck up,” convinced that Laura thought she was better than them. They were wrong on all counts. Laura's dad was a miner at the nearby colliery and her mum worked a service job. Laura just stood out because she was a twin, studious, and her parents allowed their daughters to pursue hobbies different to the hobbies of other students at her school.

I was interviewing Laura in London, where she now lives. Unbeknownst to us, we were from the same village and attended the same secondary school several school years apart. We made connections to mutual acquaintances and joked about the idiosyncrasies of our home village, exchanging memories of strange occurrences that, we proposed, could only happen there: the incompetence of the teachers, their disinterest in stopping the terrible behavior, certain characters. Laura does not carry the bullying with her in everyday life. In some ways, she is thankful for it. It forced her to leave the village as soon as possible—shortly after leaving school at 16 years old. Laura goes back from time to time to visit her parents but does not venture out to any of the local shops or pubs. She does not feel as if she belongs there.

A few days before the interview with Laura, a social media group had been unexpectedly created specifically for my school year. The group started out as a humorous space as more and more people were added. Comment threads were started remembering unrequited loves and lusts, favorite teachers, least favorite teachers, funny things that happened. People delighted as partially forgotten events or nuances were recalled: nicknames, sayings, fights, clothes, haircuts. Recollections evoked further memories in others in a collaborative remembering of who went out with who, what outrageous thing someone did, what sport they were good at, and so forth. Replies sought to convey the embodied and emotional evocations that remembering had affected, laughter and warmth, embarrassment and discomfort, all imbued with nostalgia. Affective reactions were impulsive, and expressions were presented as unproblematic (Hoskins, 2018; Pasupathi and Wainryb, 2018; Wang, 2018). People responded to others' recollections, proclaiming how they had uninvitedly evoked sensations. The group could smell the glue in technology classrooms, hear the school bell and teachers shouting their familiar chastisements, feel the itchiness of the polyester uniform and the cheap hair gel that the boys soaked into their hair every morning. Soon enough, copies of photographs were being posted, group members grimacing in horror at the sight, posting emoticons to express their emotional responses.

Transportation back to that time and place rendered a romantic yearning among lives continuously becoming, still precarious and in flux. Like in the previous vignette, the absence of the coal industry, and other forms of industrial employment, as a post-education route, and neoliberal employment in their place, had fragmented our trajectories. And, from people posting about their post-school lives, it was clear that there were varying degrees of disconnection, uncertainty, and insecurity. School life seemed like a safer place to return to than the unknown future. A fond place to anchor communal temporal belongings.

It was unnerving then when the online atmosphere transformed from good-natured to more problematic themes. Comments began appearing from individuals who still carry resentments from their school years. They intended to correct the nostalgic narrative that was forming, seen as a distortion of what school was really like. Many others followed, sharing their own traumatic memories of bullying, name calling, physical violence, and abuse. The dominant memory for these former students was that they could not wait to leave that school, that those years had scarred them in ways still apparent. The group suddenly fell silent, comments were no longer forthcoming. I imagined people gripping their smartphones with apprehension, like I was. For a moment, it felt as we were all in that school again, facing a reckoning.

People began to send consoling messages to those expressing traumatic memories. Some grew anxious, worried about what bad thing someone else would remember, or, rather, what someone cannot forget, an event or feeling of exclusion that is not so much a memory, but a constant living with, a persistent unsettling presence. Absenting physical violence, at times I engaged in the exclusionary practices being documented by my peers. I am bound up in the traumatic experiences of others in my year group and my younger and present self appears in affective memories of those experiences. Outside the online group, my friends and I reassured each other that we were not as bad as we individually might think we had been. We remembered much worse things perpetrated by other individuals, people we now realize had much worse historic adolescent family situations than our own.

Although I had reasonably fond memories of school, I also remember the anxiety, necessity and struggle to belong, as we all did. There were very few strategies available to do this (Bright, 2011). Some carved out their place through sport, being attractive, or being funny. No one achieved a sense of belonging by being discernibly clever (Ingram, 2011). Belonging was sacrificed by those who sought academic achievement and earmarked them for bullying, as did, seemingly, anything else. Difference was policed, and normative embodied performances instilled, not because dominant groups were so sure and certain of themselves, but because they were not. We were all anxious to get by in ways available to us and deflected attention from our own struggles and anxieties by placing it on more disadvantaged or vulnerable others. It is only since leaving school that former students from the year group have begun to make the correlations between many of those that were bullied and the poverty and hardships that they endured at home.

These lived historicities problematize nostalgic memories of school and adolescence. To what extent you can blame the behavior of teenagers on the teenagers themselves is negligible. I could, of course, be seeking to absolve myself for past behavior, to reconcile memories. I do not want to use deindustrialization or social inequality as exculpations, however, they are explanatory. A few years after we left, my former school went into “special measures” and many others in the coalfield—an area of nationally low educational attainment—have been under special interventions. Like most in the Nottinghamshire coalfield, then and now, ours was a tough, low performing school at a tough, low performing time in a tough, low performing place (Gore and Smith, 2001). Our futures were forestalled then, but our declarative memories can also alienate us from place and time. Declarative memories of industrial ruination insist us, and will forever insist us, to come to terms with our deindustrialized pasts. As the collaborative remembering of the social group attests, this is a process that persists over time. This mediates productions of belonging through autobiographical memory. However, the autobiographical self is also contingent on making sense of historical narratives and historicities that predate us (Preece, 2020). From a place-specific perspective, these too are problematic in the Nottinghamshire coalfield.

## “Scratching an Itch or Salving a Wound”: Place-Histories of Nostalgia and Trauma

Lodge banners of the National Union of Mineworkers (NUM) from collieries and coalfield communities across Britain are marched to the annual Durham Miners' Gala every July to congregate in a mass demonstration and enaction of trade union solidarity, coalmining culture, and communal class belonging (Mellor and Stephenson, 2005; Wray, 2009). Now that all the collieries in Britain have closed, the contemporary Durham Miners' Gala emphasizes the cultural and class heritage of mining and community as a commemorative act of strength and endurance. When the majority of the Nottinghamshire coalfield's miners continued working during the 1984–85 Miners' Strike and subsequently left the NUM to form the Union of Democratic Mineworkers, they were forever banished from the Durham Miners' Gala. Conversely, since 1985 the Nottinghamshire coalfield's striking miners have received a special welcome at the Durham Miners' Gala in acknowledgement of their solidarity despite marginalized status in their own coalfield (Wray, 2009; Emery, 2018).

When my wife and I attended the 134th Durham Miners' Gala in the summer of 2018 we walked alongside an NUM branch from the Durham coalfield. When their brass band played *Gresford*, the Miners' Hymn, tears ran uncontrollably down my wife's face. Her maternal family are from the Durham coalfields. Her grandfather was a staunch striker during the 1984–85 Miners' Strike. The tears were triggered by both pride and mourning. She grew up with intergenerational narratives of the strike and her family's role in it. Much like other striking families I have interviewed, the 1984–85 Miners' Strike is a part of their everyday lives and used to contextualize and explain many local and national sociopolitical developments, particularly the decline of local mining communities. The strike and its constant remembrance plays a critical role in the construction and foundation of who these families collectively are and have been.

Throughout our day, I could not shake a sense of shame. I am from a scabbing family, both on my dad's side and my mum's. I am not wholly proud of my own place histories, and shameful of the role that family and place ancestors played in the 1984–85 Miners' Strike and wider narratives of deindustrialization and trade union history. While this research has helped me understand why miners from my home place and own family did not strike in the 1984–85 Miners' Strike, I would prefer them to have done and envy my peers from striking families who hold a sense of pride from this ancestry (Wray, 2009; Bright, 2016). With an increased historical knowledge—gained from a wider research project—evocations of this place-history, inescapably *my* place-history, seem to leap out from landscapes, books, songs, films, and so forth, that previously laid anonymous and taken-for-granted.

On my drive into the Nottinghamshire coalfield I pass through Ollerton, a former mining village. There is a bench there that commemorates David Jones, a striking miner from Yorkshire killed in March 1984 during running battles between police, pickets and working miners. Every March, David Jones' family and friends, come to the bench to lay flowers and post pictures of David so that Ollerton never forget his face or what occurred there that night. The bench's inscription reads that David “lost his life at Ollerton … in the fight to save jobs and communities.” When I approach this unassuming bench, I cannot help but look, and seeing it unavoidably evokes feelings of shame and alienation from histories that I belong to and am entwined with.

Previously, I might have passed David's bench hundreds of times and not given it a thought. Neither did Gary and Sarah and they are from Ollerton. Their dads spoke “all the time” about their mining days and Sarah's dad often mentions how the colliery closure “killed the village.” The logo of Ollerton Town Council, like others in the coalfield, features a colliery headstock and Gary and Sarah both spent much of their childhoods in the now demolished Miners' Welfare building with other mining families. Gary “used to have a great time in there” and the Miners' Welfare was where Sarah learnt to dance and met her best friend. Yet neither Gary or Sarah knew much about the 1984–85 Miners' Strike, the violence that ripped through their village during those 12 months, or the bitter cleavages left in its wake.

I realize now that our ignorance resulted from a sustained and ongoing process of silencing and social forgetting (Hirst and Yamashiro, 2018). Some Ollerton residents objected when the bench was proposed, arguing that it should be placed in Yorkshire where David was from. My historical geographies, along with Gary's and Sarah's, and others, were primarily presented to me in ways that my family and older generations wanted them to be, as nostalgic recollections of a time that we should mourn. When I asked friends and participants what they know about their shared place-histories they were convinced they were fully aware of their parents' and grandparents' lives, that memories were always passed down to them, that they knew where they were from through these stories. Though, apart from members of striking families, they did not know about the 1984–85 Miners' Strike. They did not know about the split in the unions or know much about colliery closures or the contextual underpinnings of our deindustrialized lives. In particular, the 1984–85 Miners' Strike in the part of the Nottinghamshire coalfield we are from takes the form of a collective place secret, lingering underneath, but never explicitly spoken of.

On the odd occasion that anyone from the deindustrialized generation reflected on or questioned things transferred to us, we filled in the interstices with assumptions and inferences. Laura decided to write a play about the Nottinghamshire coalfield inspired by conversations with her father. She knew of the 1984–85 Miners' Strike but did not want to write a play about it, believing that no one would want to watch it. It was only from delving into the history of the strike that she realized the significance of the industrial unrest to the area and the wider story of deindustrialization and changed her mind. When Laura told her parents that the play was to be about the strike, her mum warned her off. Laura had one conversation with her father on the phone about the strike. The conversation developed into an argument and Laura's father got so agitated and overflowing with emotions, that he put the phone down on his daughter. He could not talk about any aspect of the strike without transgressing his own boundaries of emotional expression, so they never spoke of it again. Like Laura, my curiosity into the place I am from has also led to awkward interactions with family and family friends. Ex-miners and wives of ex-miners have started slipping comments about the 1984–85 Miners' Strike and colliery closures into conversations I am within earshot of, comments that they see as exonerating them for past actions and decisions. People have said such things as, “Well, you never hear about the Strike from our side, do you? How we were lied to.” Others have suggested that they were excused from going on strike because they were in other unions within the industry and that they were critical to maintenance of the colliery.

The feeling that exposing concealed wounds compounds the confusing sense that I am being alienated from my own place by knowing more about its past. Walley's (2013, p. 22) metaphor of the need to find out and to understand the causes of industrial ruination being like “scratching an itch, or salving a wound” resonates with me, but any catharsis that scratching the itch has rendered remains unclear. It often feels like in scratching the itch I have broken the skin and the wound has been infected with a bitterness and alienation that was not previously felt so intensely.

## Discussion

The above vignettes articulate (some of) the affective-temporal processes operating in spaces, atmospheres, materialities, and bodies with contested pasts and deindustrialized presents. We have seen how intergenerational transmissions, declarative memories, and place-histories are enacted in shared and personal spaces, bodies, and emotions (and this should not be seen as the extent of the myriad temporal processes that intervene in everyday relations in deindustrialized spaces and elsewhere). As we navigate everyday becomings through life courses we encounter affective-temporal processes of declarative memory, place histories, and intergenerational transmissions, each shaping how the others are lived, felt, and transformed. Affective-temporal processes relate, overlap and pile-up (Stewart, 1996). The ways that relational affective-temporal processes emerge and are enacted in formations of belonging and alienation is further contingent on spatial and political contexts of their emergence in the present. These relational encounters, in turn, condition and mediate our attempts and capacities to belong and can alienate us from space and time.

I have contributed to understandings of how past formations of belonging are transmitted and emerge in a deindustrialized generation that had little direct experience of the industries that ordered previous forms of life and place (Nayak, 2003; Walkerdine and Jiménez, 2012; Bright, 2016; Walkerdine, 2016). Coal mining, the coal industry and mining communities in the Nottinghamshire coalfield, that the generation examined in this article knew only as residues and legacies, are traceable in practices, performances, and desired means to belong that emerge in different moments, encounters, and contexts. During the period when coal dominated the socioeconomic and cultural rhythms of the coalfield, intergenerational social reproduction of belonging and identity for men were given clear avenues by the availability of mining jobs, whereby sons of miners could become miners themselves. These affective and embodied ways of being have transferred to a generation that are bereft of the institutional and economic systems and infrastructure to claim belonging in what we see as our legitimate prideful and purposeful heritage. Without mining jobs, formulations of valued work and employment coheres around subsidiary characteristics of physical labor, defined and evidenced by how that work inflicts itself on the body.

Affective intergenerational transmissions do not inform the totality of how people seek to claim, and are denied, belonging among this generation, however. While industrial mining cultures and forms of life continue to make themselves known in discreet and subtle ways, shared experiences of industrial ruination and neoliberal regeneration have created a localized and classed systems of knowing. This spatially and historically specific knowledge is rendered contingent of the historicities of deindustrialized bodies enmeshed in the Nottinghamshire coalfield. Articulating and describing localized systems of knowing proves difficult and we often distil meanings into phrases dense with spatial and embodied intensities, such as “typical Mansfield lad.” Inarticulable is the mutuality of deindustrialized spaces, temporalities, and people to co-produce the atmospheres, moods, and feel of places and the types of bodies that occupy them. Disdained bodies and atmospheres simultaneously alienate, exclude, and draw in.

These deindustrializing affective conditions are bound up in the process of lived memories, whereby declarative affective memories of living deindustrialization have begun to problematize the perceptions and imaginaries of the past. This is the case of my school year's social media group. Here, the effects of deindustrialization, for instance, poverty, social inequalities, and familial breakdowns led to difficult experiences of school where bullying and anxiety was endemic. Negatively valenced affective memories of this time contest nostalgic evaluations of the past. Moreover, this contested nostalgia, which could provide a resource to cope with present-day anxieties and precarities, inhibits a temporal belonging through coherent autobiographical memories. Alluding to localized specificities of place, as my generation grow older, we are forced to reconcile memories of industrial ruination with merely fragments of how things came to be.

Importantly, intergenerational transmissions are transformed and obfuscated by the geographical specificities of the Nottinghamshire coalfield's experience of deindustrialization, namely the 1984–85 Miners' Strike and trade union splits. A deindustrialized generation have been bereft of many proudful associations with our histories because of the inabilities of older parents and so forth to communicate useable pasts. Pluralizing forms of temporal processes allows us to consider what histories and historicities are being undeclared or emerge through other forms beyond speech, an undeveloped area of class research, which tends to focus on the clearly apparent and representational (Emery, 2019b). Coal mining culture and heritage is not as palpable in the Nottinghamshire coalfield as that demonstrated by the villages proudly parading at Durham Miners' Gala, for instance (Emery, 2020). Traumatic histories of the 1984–85 Miners' Strike, and its contextualization of subsequent colliery closures, are actively silenced in the Nottinghamshire coalfield resulting in an absence of explanatory frameworks for the industrial ruination experienced by my generation. The participants and friends I spoke with who are from mining families that worked during the strike had little idea of the fractious and violent events that took place in the mining villages they were from or the changes and animosities it wrought. In a similar sense to that which Bright (2012, p. 316) found in a study of the neighboring Derbyshire coalfield, a deindustrialized generation in the Nottinghamshire coalfield are “adrift from ‘illegitimate' histories that are [our] legitimate ‘heritage' and, at the same time, [are] subject to the traumatic affective legacy of those histories.”

Moreover, both Laura and I discovered that uncovering this traumatic past and breaking the silencing that requires continual vigilance can result in unsettling and fractious conversations with family members. Conversely, the two people I spoke with that were from families that went on strike were knowledgeable about the strike and took great pride in their family's action and association. Thus, the capacity to produce autobiographical narratives from intergenerational, unlived history, and use these as a resource in which to claim belonging, is dependent on the specificities of that history and an individual's position to it.

Including autoethnography also opens the analysis to claims of egocentrism, however, I have drawn out distinctions between experiences across and between participants (Bennett, 2014b). Of course, as we have seen, I am somewhat unrepresentative of the wider deindustrialized generation because I am now aware of the fine-grained histories of the Nottinghamshire coalfield. This poses a weakness with the conceptualizations, yet, as has been argued, there does not appear to be a representative individual in the coalfield, and there is no “typical Mansfield lad.” We have seen that variegated experiences are a defining aspect of deindustrializing spaces, as the old anchors of work and place are dislodged from each other, life courses and trajectories. Deindustrialization of the Nottinghamshire coalfield has pluralized and fragmented experiences such that former “ties that bind”—the colliery, the mining community and attendant social institutions—have been almost irreparably eroded. Deindustrialization and the detachment of work and community has cast deindustrialized generations out into a neoliberalized and individualized world, leading to problematized attachments and detachments from each other and place (Beck and Beck-Gernsheim, 2002; Preece, 2020). What this research evidences further is that “it is increasingly problematic to clearly define a post-industrial working-class” (Emery, 2019b, p. 3), and I have avoided speaking in exclusively class categories here, preferring to take processual and relational understandings similar to those of Thompson (1963, p. 8) who did not see “class as a ‘structure,' nor even as a ‘category,' but as something which in fact happens in human relationships.” Besides shared childhood experiences and place history, participants suggested that there is little by way of similitude between each other and me with them. I have drawn out distinctions between my experience and that of others of a similar age, illustrating the individuated nature of embodiment and emotional experience. Of course, individuality existed before deindustrialization, but it has been institutionalized and venerated through neoliberalism and regeneration policies, which the deindustrialized generation have become through. The neoliberalization of work, for example, has forced a generation of new workers to follow diffuse avenues in the pursuit of wages and belonging, eroding collectivized senses of similitude. We saw with Simon, Stephen, and me that divergent employment and education trajectories can lead to disdain of those from similar starting points. We also saw how the affective embodiments of this disdain, alienation and shame can manifest in claims over space and resources to anchor and enact belonging, in this case, claims over Mansfield and who belongs there (Fenster, 2005).

Possessing historical knowledge also differentiates me and those belonging to striking families from most of my generation in the Nottinghamshire coalfield that remain solely exposed to the past through positive memories and affective and embodied intergenerational transmissions. Those from striking families are also not vulnerable to the shame and alienation I feel towards my own family and place history. However, they may have senses of alienation to place owing to family-centered intergenerational transmission as opposed to place-history. Additionally, my white male positionality precludes me from personally feeling—in an embodied and emotional form—what it is like, for instance, for Lauren, Claire, and Kate to encounter the masculinities of the “typical Mansfield lad.” Aside from the discussion of masculinity, generation, and place, there has not been space to draw out distinctions in how affective-temporal processes intersect with other identifications of gender, race, and sexuality to mediate belonging and alienation—another productive avenue to investigate. As previously noted, shared atmospheres and collective moods and feelings are shaped by other subjectivities.

Despite their limitations, however, foregrounding my own experiences also speaks to an ethics of representation not often discussed in work that either fuses autoethnographic accounts with interview testimony or critiques of autoethnography. This paper has documented difficult, traumatic and troubling experiences, memories, and pasts. I share in the broader socio-cultural context of deindustrialization from which they emerged. Histories of deindustrialization are carried with us, worn on and in our bodies, present in embodied performances and affective repertoires. To pretend otherwise would be an abdication of my responsibility to my research participants. During interviews it was made apparent by participants that we should share together experiences and memories, rather than me merely gathering and using their testimony. It was through conversations with research participants and friends that I decided to document autoethnographic testimony in the hope that I could offer something valuable on the intimacies of belonging and alienation. To not share my own stories alongside theirs would be a dereliction of solidarity with them.

I suggest that scholars from and with (de)industrial backgrounds rooted in families and places should stand with their participants to unashamedly communicate our personal and collective narratives in order to correct or explain some of the misrecognition of class and class politics. Through doing so we can begin to unravel the social and academic prejudices toward these demographics that compound the affective intensities of alienation and bitterness they experience, and that we should seek to ameliorate. Although deindustrialization and neoliberal regeneration has split and fragmented the life courses of those with shared backgrounds, by documenting our temporal experiences and struggles to belong in all their difficult, troubling and comforting complexities we may find that our deindustrializing lives are not as heterogenous as first appears. As the narratives shared in this article attest, similitude exists in lived, class-based, affective experiences of alienation contingent of lived, class-based, histories of deindustrialization. For better or worse, our shared lived and intergenerational pasts condition our emotional and bodily repertoires, marking us out and mediating our capacities to belong in the spaces that deindustrialization has left us. These threads of shared affective-temporal processes of belonging and alienation can form the basis for a renewal of solidarity, attenuation of alienation and a means to belong.

## Data Availability Statement

The datasets generated for this study are available on request to the corresponding author.

## Ethics Statement

The studies involving human participants were reviewed and approved by University of Leicester. The patients/participants provided their written informed consent to participate in this study.

## Author Contributions

The author confirms being the sole contributor of this work and has approved it for publication.

## Conflict of Interest

The author declares that the research was conducted in the absence of any commercial or financial relationships that could be construed as a potential conflict of interest.

## Footnotes

^1^“Lads” is a slang term for young men.
